# Platypnea-Orthodeoxia Syndrome in a Frail Elderly Patient: It's Not Always Pneumonia

**DOI:** 10.7759/cureus.115016

**Published:** 2026-08-23

**Authors:** Behzad Masood, Afzaal Aleem Khan, Furqan A Taj

**Affiliations:** 1 Cardiology, Sheffield Teaching Hospitals NHS Foundation Trust, Sheffield, GBR; 2 Acute Medicine, Russells Hall Hospital, Dudley, GBR; 3 Adult Mental Health, Mayo University Hospital, Mayo, IRL

**Keywords:** cardiac platypnea-orthodeoxia syndrome, intra-cardiac shunt, orthostatic desaturation, patent foramen ovale (pfo), platypnea-orthodeoxia syndrome, positional hypoxemia, right-to-left cardiac shunt

## Abstract

Platypnea-orthodeoxia syndrome (POS) is a rare condition characterized by positional dyspnea and arterial desaturation that worsens in the upright position and improves when supine. It is most commonly associated with intracardiac right-to-left shunting, often through a patent foramen ovale (PFO), and is frequently under-recognized. We report the case of an 84-year-old frail woman admitted with acute confusion and severe hypoxemia, initially treated for presumed community-acquired pneumonia. Despite high oxygen requirements, inflammatory markers were minimally raised, and imaging did not support an infection. A review of prior investigations revealed a PFO. Formal bedside positional oxygen assessment demonstrated reproducible orthodeoxia, confirming POS. A raised right hemidiaphragm was identified as a likely contributory factor. Given the patient’s advanced frailty, a conservative approach to management was pursued. The patient was successfully weaned off oxygen and discharged home, to be nursed in a semi-recumbent position.

## Introduction

Platypnea-orthodeoxia syndrome (POS) is defined by the combination of dyspnea and arterial oxygen desaturation in the upright position with improvement on lying supine. Although rare, it is a well-described phenomenon most commonly associated with intracardiac shunting through a patent foramen ovale (PFO) or atrial septal defect in the absence of pulmonary hypertension [1,2].

Diagnosis is frequently delayed or missed, as symptoms often mimic more common cardiopulmonary conditions. Elderly patients are particularly vulnerable to misdiagnosis, leading to unnecessary investigations and treatment. This case illustrates a typical diagnostic pitfall and reinforces the importance of positional oxygen assessment in unexplained hypoxemia.

## Case presentation

An 84-year-old woman was admitted from the community with acute confusion and hypoxemia. Community observations documented oxygen saturations of 87% on room air, improving with 2 L/min of supplemental oxygen. All other observations were within normal range, and her GCS was reported as 14 (due to confusion). Her medical history included IgG monoclonal gammopathy of undetermined significance, previous mastectomy for breast cancer, hypertension, osteoarthritis, and chronic anaemia. She was bedbound at baseline and clinically very frail.

On admission, she was significantly more hypoxic than what was noted on community observations, requiring up to 15 L/min of oxygen via a non-rebreather mask to maintain saturations above 94%. She was again noted to be confused with a GCS of 14/15. She was initially started on treatment for a presumed community-acquired pneumonia, with intravenous antibiotics.

**Figure 1:**
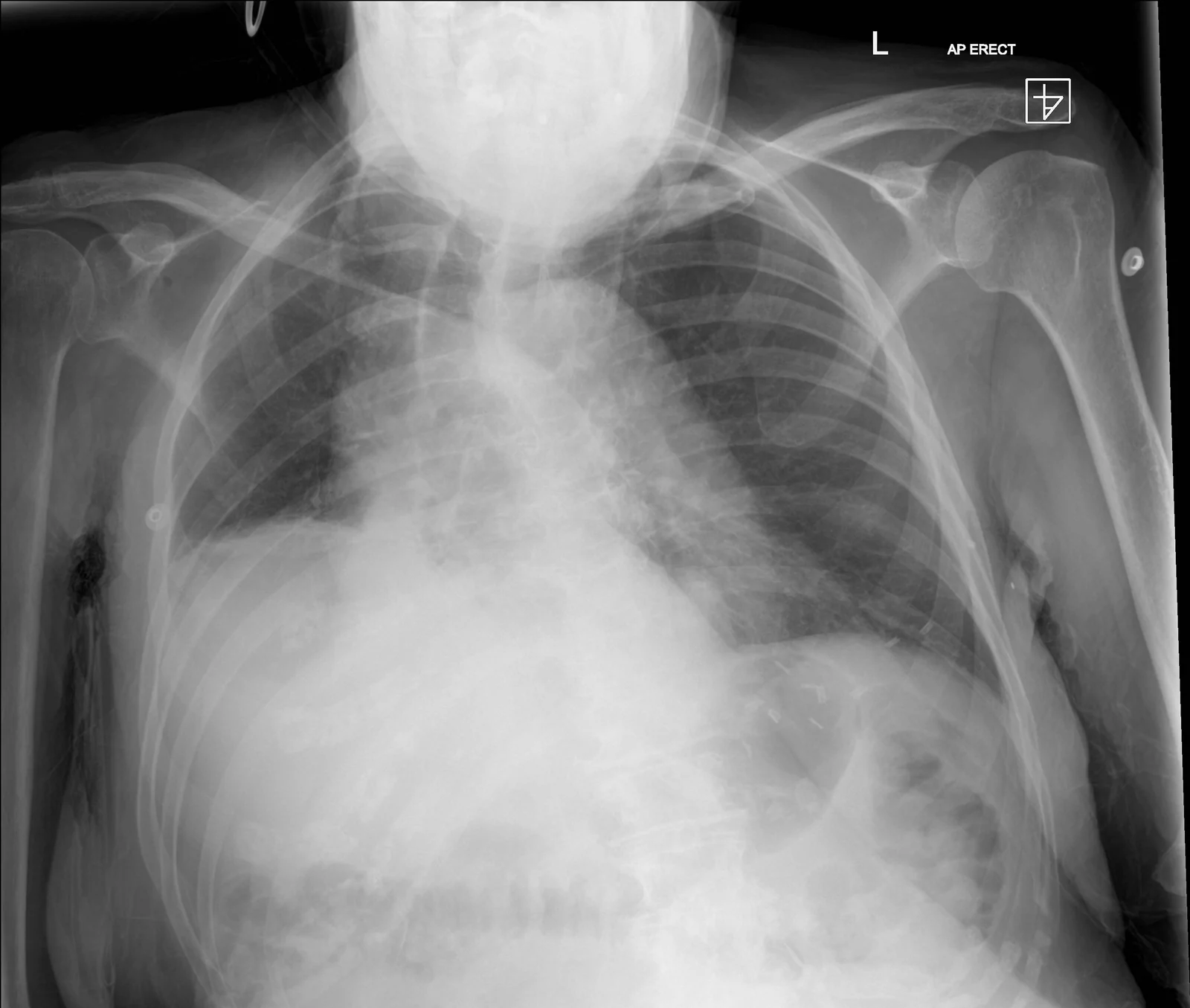
Chest X-ray showing a raised right hemi-diaphragm

Chest radiography done on admission demonstrated elevation of the right hemidiaphragm without focal consolidation, as shown in Figure 1. Laboratory investigations showed a C-reactive protein of 7 mg/L and a white cell count of 11 × 10⁹/L. There were no recorded febrile episodes, and procalcitonin was negative. Relevant laboratory investigations are given in Table 1.

**Table 1:** Laboratory findings and reference values CRP, C-reactive protein; WBC, white blood cell count; PCT, procalcitonin

Laboratory parameter	Patient value	Reference value
C-reactive protein (CRP), mg/L	7	<6
White blood cell count (WBC), ×10⁹/L	11	4–11
Procalcitonin (PCT), ng/mL	0.10	<0.25

Clinical examination revealed reduced air entry at the right lung base. Cardiac examination was unremarkable, with no murmurs.

On senior review, prior investigations were revisited, including a transthoracic echocardiogram with agitated saline contrast that had previously demonstrated a PFO. In view of this finding and the marked discrepancy between oxygen requirements in the community and in hospital, POS was suspected.

A positional oxygen assessment was performed. While seated upright on 15 L/min oxygen via a non-rebreather mask, oxygen saturations were 94-95%. Removal of supplemental oxygen in the upright position resulted in desaturation to 83-84% on room air. When placed supine off oxygen, saturations improved to 89-91%. Re-sitting the patient upright on a 60% Venturi mask resulted in saturations of 85-88%, which again improved to 94-95% when returned to the supine position on the same oxygen concentration. Oxygen saturation was monitored continuously during each positional change and recorded once a stable plateau had been reached. The positional response was reproducible on repeat assessment.

These findings confirmed reproducible orthodeoxia, which is defined as a fall in oxygen saturation of >5 percentage points. In conjunction with the known PFO and exclusion of alternative causes of hypoxaemia, a diagnosis of POS was made, and the intravenous antibiotics were stopped. Given the patient’s advanced frailty and bedbound status, invasive intervention was not considered appropriate. Conservative management was advised, including nursing in a semi-recumbent position and consideration of long-term oxygen therapy if weaning was unsuccessful. The patient was subsequently successfully weaned off oxygen in the hospital and discharged home, to be nursed in a semi-recumbent position.

## Discussion

POS is most commonly caused by intracardiac right-to-left shunting through a PFO, typically in the presence of additional anatomical or functional factors that alter atrial geometry or venous flow [2,3]. Although PFOs are common, their presence alone does not cause POS. Under normal circumstances, the PFO remains functionally closed and does not produce clinically significant right-to-left shunting. In case of POS, however, the PFO provides the anatomical communication, whereas a second anatomical or physiological factor creates a posture-dependent shunt. On assuming the upright position, gravitational changes in venous return and cardiac orientation may alter the relationship between the right atrium, interatrial septum, and caval inflow. Deoxygenated blood may then be preferentially directed from the inferior vena cava towards the PFO, the PFO tunnel may be stretched open, or right atrial pressure may transiently increase relative to left atrial pressure. Relevant contributing factors include aortic elongation or dilatation, atrial septal aneurysm, kyphoscoliosis, pneumonectomy or a diaphragmatic elevation [4,5].

In this patient, a raised right hemidiaphragm was considered the possible additional anatomical factor. It may have altered intrathoracic geometry, right atrial orientation, or caval venous flow, thereby favouring right-to-left shunting across the PFO in the upright position. Similar cases of POS associated with extracardiac triggers, including hemidiaphragmatic elevation or paralysis, have been reported in elderly patients, in whom age-related anatomical changes may unmask previously asymptomatic shunts [3,6,7]. However, because posture-dependent shunting was not directly visualized with positional transoesophageal echocardiography or invasive haemodynamic assessment, the raised hemidiaphragm should be regarded as a plausible contributing factor rather than a definitively established cause.

This patient’s initial presentation was attributed to pneumonia; however, minimally raised inflammatory markers, absence of fever, negative procalcitonin, and lack of radiographic consolidation made infection unlikely. The dramatic positional variation in oxygenation proved to be the key diagnostic clue. This case highlights the importance of reassessing the working diagnosis when clinical features do not align with investigation results.

Definitive treatment of POS usually involves percutaneous closure of the PFO, which is associated with rapid and sustained improvement in oxygenation and symptoms [3]. However, in frail or bedbound patients, the risks of intervention may outweigh the benefits, and conservative management may be more appropriate.

Limitations

This case report has some limitations. The diagnosis was based on reproducible positional pulse oximetry, a previously documented PFO, and the available clinical, laboratory, and imaging findings. However, positional transoesophageal echocardiography or cardiac catheterisation was not performed to directly demonstrate or quantify posture-dependent right-to-left shunting, owing mainly to the advanced frailty of the patient. Although the raised right hemidiaphragm was considered a possible anatomical contributor, its causal role was not definitively established by dedicated assessment of diaphragmatic function or positional changes in cardiac geometry; this, again, was not attempted due to the patient's frailty, i.e., to avoid unnecessary investigations that would not change the management.

## Conclusions

Platypnea-orthodeoxia syndrome (POS) is an uncommon but important cause of positional hypoxemia that is frequently misdiagnosed. Simple bedside positional oximetry, combined with careful review of prior investigations, led to the correct diagnosis for this patient, without the need for invasive investigations. This case underscores the need to consider POS in patients with unexplained or disproportionate hypoxemia and demonstrates the importance of individualized management decisions in frail patients.
